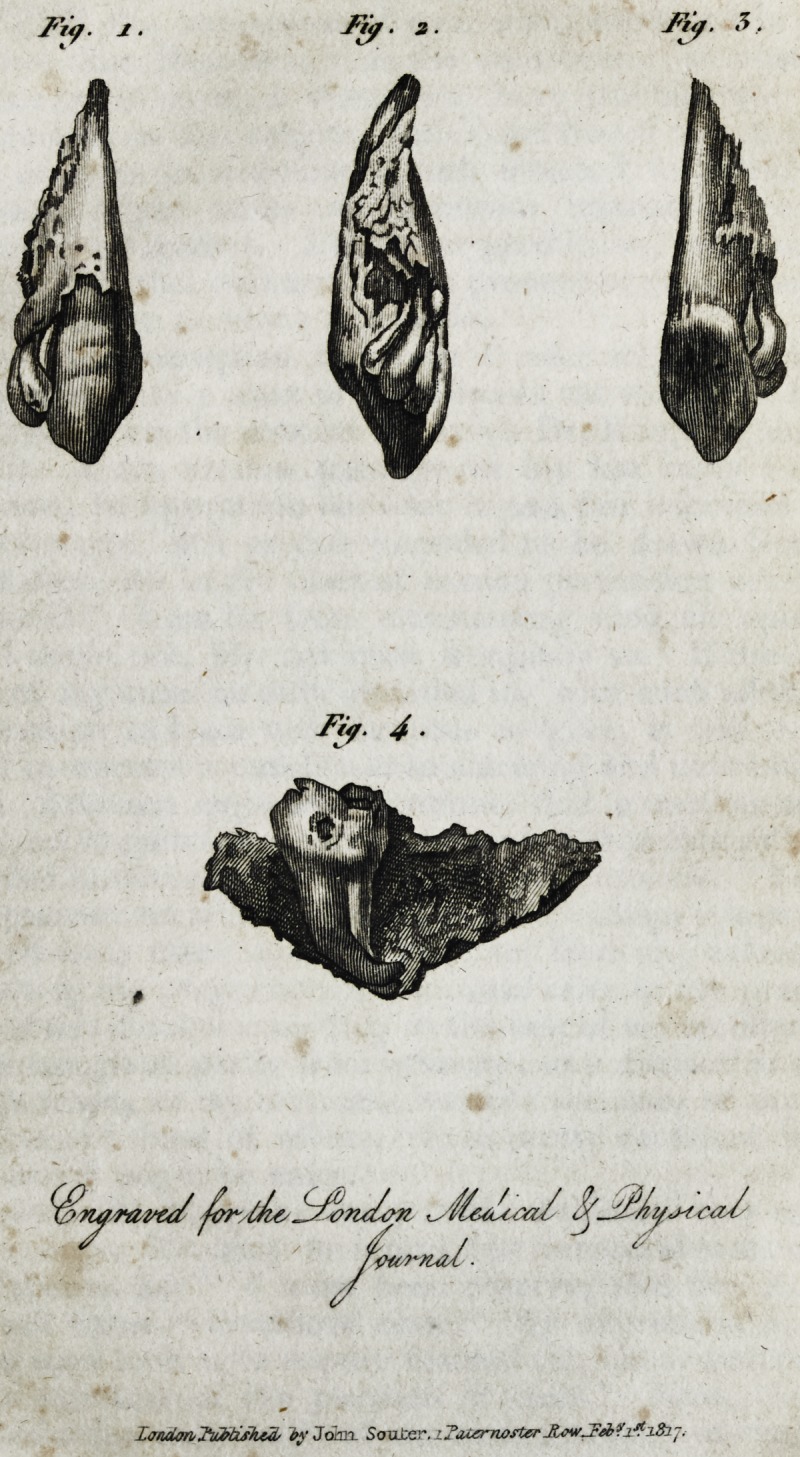# On a Peculiarity in the Structure of the Teeth

**Published:** 1817-02

**Authors:** 

**Affiliations:** Surgeon-Dentist.


THE LONDON
### Medical and Physical Journal.

2 OF VOL. XXXVII.] FEBRUARY, 1817. [NO. 216.

"For many fortunate discoveries in medicine, and for the detection of numerous errors, the world is indebted to the rapid circulation of Monthly Journals; and there never existed any work to which the Faculty in EUROPE and AMERICA were under deeper obligations than to the Medical and Physical Journal of London, now forming a long, but an invaluable, series."—RUSH.

*For the London Medical and Physical Journal.*

### *On a Peculiarity in the Structure of the Teeth;* by Mons. LEMAIRE, Surgeon-Dentist.

ABOUT two years since, a gentleman came to me with his daughter, aged about sixteen; her teeth were very beautiful; the upper lip on the right side was much more elevated than on the left, which led me to suppose, that he wished to consult me on an excrescence formed on the gums; but, on examining, I found that the temporary cuspidatus had been extracted to make room for the secondary incisores, as is now the common practice. The first small molaris had replaced the cuspidatus in such a manner, that the latter, meeting with obstacles, had not shown itself between the first little molaris and the little incisores, until the young lady had reached her sixteenth year. It was just visible when I was consulted. I deferred the extraction for a month. Many dentists would have extracted the first little molaris, in order that the cuspidatus might take its place; but it is a principle with me never to derange what appears to be well, and I always say to myself—*when in doubt act not.*

Six weeks afterwards, the young lady was brought to me again; the corona of the cuspidatus was partially shot forth; the gum was much swelled and painful. In raising it lightly with the probe, I perceived, on the lateral posterior surface, a body which I at first took to be tartar, being precisely of the same colour; I attempted to remove it previous to the extraction of the tooth. This, perceiving the adherence was very strong, I abandoned, and, with my instrument reversed, I made a demi-luxation, and afterwards terminated the operation by extracting the tooth downwards, without causing much pain or lacerating the gum. My surprize was extreme, when, instead of finding *one* cuspidatus, I found *four*, very distinct, detached from each other, and undoubtedly produced by four different germs. I showed the piece to

Drs. Majendie, Breschet, and Beclard, who wished me to have it designed and engraved, which I should probably not have done, if the work of Mr. Fox had not fallen into my hands. This quarto volume, printed in a most expensive style, contains nineteen plates on the vicious formation of the teeth, the disorders of the gums and jaws, &c.; which have been already long and well known, and it is evident that the author had never met with a case similar to the present one. In fact, they must be extremely rare, and, perhaps, the one now presented is unique; be this, however, as it may, the subject is calculated to exercise the imagination of our physiologists.

*Paris; Dec.* 1816.

*Explanation of the Plate.*—Fig. 1, represents the external surface;—2, the lateral and posterior ditto;—3, the posterior ditto; —4, is the result of an ill-understood operation, practised too frequently, even at Paris. An unfortunate patient, aged thirty-six, in the eighth month of her pregnancy, went lately to a dentist to have a tooth extracted, which had appeared at the age of twenty, commonly called the wise tooth. She had already lost three of the molares (two large, and one small,) on the same side. The tooth in question, quite isolated by this loss, had a considerable caries in the inside, but, on the outside, afforded ample space for attaching the forceps. The dentist wished to extract it inwards, which ought never to be done, except when the teeth are loose. In that case we do not risk the fracture of the jaw, as was the case in the present instance; and, had I not been called in in time, the unfortunate woman would have inevitably perished. The commotion was so strong, and the buccinatores muscles and a portion of the constrictor superior of the pharynx so torn, that a dreadful hæmorrhage was the consequence, which prevented my extracting this monstrous splinter for twenty-four hours. The swelling was prodigious, the patient could not swallow even liquids; I experienced great difficulty in the operation of separating the splinter (which was one inch and three-quarters long, and six-tenths of an inch high) from the tooth; the adherence was so strong, that they appeared to form only one body. The extraction of this body, lemonade in abundance, lavemens, and bathing the feet, in a few days placed the patient out of danger. And may the example teach dentists to beware of following old systems, disavowed by nature and science, and rather decline a hazardous operation than risk the lives of those who honor us with their confidence.

---

We believe the French were the first to improve that part of surgery which regards the beauty of the teeth: but, in this, as in most other instances, they are much behind us in pathology. We trust the above, which we received from Paris, will attract the notice of some of our London surgeon-dentists, from one of whom we were some time ago promised a communication.—EDIT.

## Figures and Tables

**Fig. 1. Fig. 2. Fig. 3. Fig. 4. f1:**